# Supplementary material for: Spurious early ecological association suggesting BCG vaccination effectiveness for COVID-19
Source: PLoS One. 2022 Sep 20;17(9):e0274900. doi: 10.1371/journal.pone.0274900 (PMC9488757; doi:10.1371/journal.pone.0274900)
Supplement: Attachment — Submitted filename: Response to Reviewers (1).docx [file pone.0274900.s002.docx]

**Journal requirements**

**Please ensure that your manuscript meets PLOS ONE's style requirements, including those for file naming.**

We have updated our manuscript such that it meets PLOS ONE’s requirements.

**Please state what role the funders took in the study. If the funders had no role, please state: "The funders had no role in study design, data collection and analysis, decision to publish, or preparation of the manuscript." If this statement is not correct you must amend it as needed. Please include this amended Role of Funder statement in your cover letter; we will change the online submission form on your behalf.**

The following statement is correct:

“ML and PL receive funding from the Sydney E. Frank Foundation. The funder had no role in study design, data collection and analysis, decision to publish, or preparation of the manuscript.”

This has also now been appended to the cover letter.

**Please note that funding information should not appear in the Acknowledgments section or other areas of your manuscript. We will only publish funding information present in the Funding Statement section of the online submission form. Please remove any funding-related text from the manuscript and let us know how you would like to update your Funding Statement.**

We no longer have any Acknowledgements.

**We note that you have stated that you will provide repository information for your data at acceptance. Should your manuscript be accepted for publication, we will hold it until you provide the relevant accession numbers or DOIs necessary to access your data. If you wish to make changes to your Data Availability statement, please describe these changes in your cover letter and we will update your Data Availability statement to reflect the information you provide.**

We have now included the underlying data in complete form as a supporting information CSV file. We have included the following amendment in the cover lever in regards to Data Availability:

“All data used in this analysis is available in the supporting information section as File S1.”

**Comments to the author**

**Is the manuscript technically sound, and do the data support the conclusions?**

**The manuscript must describe a technically sound piece of scientific research with data that supports the conclusions. Experiments must have been conducted rigorously, with appropriate controls, replication, and sample sizes. The conclusions must be drawn appropriately based on the data presented.**

**Reviewer #1: Yes**

**Reviewer #2: Partly**

**Has the statistical analysis been performed appropriately and rigorously?**

**Reviewer #1: Yes**

**Reviewer #2: No**

We would like to clarify that this manuscript is not seeking to establish the efficacy of BCG for COVID-19 using the Miller methods. Rather, we are seeking to examine the appropriateness of their methods. Our hypothesis was that the apparent association would not stand the test of time, and generally that hypothesis was upheld. We conclude with warnings about the hazards of using ecological data to assess product efficacy, particularly in the context of a rapidly moving pandemic.

**Have the authors made all data underlying the findings in their manuscript fully available?**

We have now included the underlying data as a supporting information CSV file (S1 File).

**Is the manuscript presented in an intelligible fashion and written in standard English?**

**Reviewer #1: Yes**

**Reviewer #2: Yes**

No response necessary

**Review Comments to the Author:**

**Reviewer #1: This is a useful contribution to the literature.**

**In the Conclusions, the authors frame the value of ecologic studies as hypothesis generating and findings should be interpreted with caution.**

**Ecologic studies certainly have value and indeed may be the most appropriate data for helping to answer certain questions (eg the impact of policy changes on population level outcomes). I would suggest moderating these claims.**

Thank you for your thoughtful review of the manuscript. We agree that ecologic studies can be beneficial for assessing policy changes, generating hypotheses, and examining population-level temporal trends. We have now included an additional paragraph in the Discussion section (lines 177–184) outlining these benefits:

“Ecologic studies have provided valuable initial understandings of the effects of various exposures on population-level disease outcomes. Innovative ecologic studies of lactation on breast cancer^1^, salt intake and blood pressure^2^, and male circumcision on HIV transmission^3^, have provided important insights into causal hypotheses at the individual-level. Other advantages of these studies includes the ability to efficiently examine trends over time, similar to the analysis here where the BCG-COVID relationships were reassessed as the pandemic progressed. Further, ecologic studies are a critical part of the methodological toolbox for evaluating the impact of policy changes and interventions by examining pre and post population-level outcomes.”

**Reviewer #2: Although authors referred to the Miller´s paper as the main point for the analysis, there are several other reports to be taken in consideration that authors omitted. Then a paper with only 14 references for such an important topic. It would recommended to work on those papers to enrich the discussion and conclusions.**

Thank you for your detailed review of the manuscript. We have now referenced several similar works to strengthen the Discussion section. The following is the additional paragraph we have now included to discuss other ecologic studies (lines 202–209):

“Subsequent ecologic studies of the relationship between BCG vaccination and COVID-19 attempted to overcome these biases. For example, several studies attempted to account for potential exposure misclassification by using vaccine coverage rather than policy as the exposure of interest^4–13^. Others used multivariable frameworks to account for confounding^4,7,8,10,12–20^, but important confounding variables such as health care access, health care quality, and health consciousness were typically not addressed. Moreover, potential differential outcome misclassification bias, coexistence of COVID-19 mitigation policies, and the dynamically changing nature of the pandemic remain and have yet to be fully accounted for in these analyses. ”

**References**

1.	MacMahon B, Lin TM, Lowe CR, Mirra AP, Ravnihar B, Salber EJ, et al. Lactation and cancer of the breast. A summary of an international study. Bull World Health Organ. 1970;42(2):185–94.

2.	Stamler J. The INTERSALT Study: background, methods, findings, and implications. The American Journal of Clinical Nutrition. 1997 Feb 1;65(2):626S-642S.

3.	Wamai RG, Morris BJ, Bailis SA, Sokal D, Klausner JD, Appleton R, et al. Male circumcision for HIV prevention: current evidence and implementation in sub-Saharan Africa. J Int AIDS Soc. 2011 Jan;14(1):49–49.

4.	Escobar LE, Molina-Cruz A, Barillas-Mury C. BCG vaccine protection from severe coronavirus disease 2019 (COVID-19). Proc Natl Acad Sci U S A [Internet]. 2020;117(30):17720–6. Available from: http://www.ncbi.nlm.nih.gov/pubmed/32647056

5.	Mariita RM, Musila JM. A study on the relationship between Bacillus CalmetteGurin (BCG) vaccination and Covid-19 prevalence: Do other confounders warrant investigation? Journal of Public Health and Epidemiology. 2020 Jun 30;12(2):142–50.

6.	Mohamed Hussein AAR, Salem MR, Salman S, Abdulrahim AF, al Massry NA, Saad M, et al. Correlation between COVID-19 case fatality rate and percentage of BCG vaccination: is it true the vaccine is protective? The Egyptian Journal of Bronchology. 2020 Dec 9;14(1):25.

7.	Ogimi C, Qu P, Boeckh M, Bender Ignacio RA, Zangeneh SZ. Association between live childhood vaccines and COVID-19 outcomes: a national-level analysis. Epidemiology and Infection. 2021 Mar 16;149:e75.

8.	Li WX. Worldwide inverse correlation between Bacille Calmette–Guérin (BCG) immunization and COVID-19 mortality. Infection. 2021 Jun 25;49(3):463–73.

9.	Lerm M. On the relationship between BCG coverage and national COVID‐19 outcome: could ‘heterologous’ herd immunity explain why some countries are better off? Journal of Internal Medicine. 2020 Dec 14;288(6):682–8.

10.	Urashima M, Otani K, Hasegawa Y, Akutsu T. BCG Vaccination and Mortality of COVID-19 across 173 Countries: An Ecological Study. International Journal of Environmental Research and Public Health. 2020 Aug 3;17(15):5589.

11.	Kinoshita M, Tanaka M. Impact of Routine Infant BCG Vaccination on COVID-19. Journal of Infection. 2020 Oct;81(4):625–33.

12.	Wickramasinghe D, Wickramasinghe N, Kamburugamuwa SA, Arambepola C, Samarasekera DN. Correlation between immunity from BCG and the morbidity and mortality of COVID-19. Tropical Diseases, Travel Medicine and Vaccines. 2020 Dec 28;6(1):17.

13.	Klinger D, Blass I, Rappoport N, Linial M. Significantly Improved COVID-19 Outcomes in Countries with Higher BCG Vaccination Coverage: A Multivariable Analysis. Vaccines (Basel). 2020 Jul 11;8(3):378.

14.	Berg MK, Yu Q, Salvador CE, Melani I, Kitayama S. Mandated Bacillus Calmette-Guérin (BCG) vaccination predicts flattened curves for the spread of COVID-19. Science Advances. 2020 Aug 7;6(32).

15.	Brooks NA, Puri A, Garg S, Nag S, Corbo J, Turabi A el, et al. The association of Coronavirus Disease-19 mortality and prior bacille Calmette-Guerin vaccination: a robust ecological analysis using unsupervised machine learning. Scientific Reports. 2021 Dec 12;11(1):774.

16.	Ebina-Shibuya R, Horita N, Namkoong H, Kaneko T. Current national policies for infant universal bacille Calmette-Guérin vaccination were associated with lower mortality from coronavirus disease 2019. Clinical and Experimental Vaccine Research. 2020;9(2):179.

17.	Ricco’ M, Ranzieri S. BCG vaccination and COVID-19: Was flattening the curve just an illusion? Infectious Diseases Now. 2021 Aug;51(5):495–7.

18.	Hensel J, McAndrews KM, McGrail DJ, Dowlatshahi DP, LeBleu VS, Kalluri R. Protection against SARS-CoV-2 by BCG vaccination is not supported by epidemiological analyses. Scientific Reports. 2020 Dec 27;10(1):18377.

19.	Gong W, Wu X. Is the tuberculosis vaccine BCG an alternative weapon for developing countries to defeat COVID-19? Indian Journal of Tuberculosis. 2021 Jul;68(3):401–4.

20.	Joy M, Malavika B, Asirvatham ES, Sudarsanam TD, Jeyaseelan L. Is BCG associated with reduced incidence of COVID-19? A meta-regression of global data from 160 countries. Clinical Epidemiology and Global Health. 2021 Jan;9:202–3.
